# A Mobile App (FallSA) to Identify Fall Risk Among Malaysian Community-Dwelling Older Persons: Development and Validation Study

**DOI:** 10.2196/23663

**Published:** 2021-10-12

**Authors:** Devinder Kaur Ajit Singh, Jing Wen Goh, Muhammad Iqbal Shaharudin, Suzana Shahar

**Affiliations:** 1 Center for Healthy Ageing and Wellness Faculty of Health Sciences Universiti Kebangsaan Malaysia Kuala Lumpur Malaysia; 2 Faculty of Health Sciences Cawangan Pulau Pinang, Kampus Bertam Universiti Teknologi Majlis Amanah Rakyat Penang Malaysia

**Keywords:** fall risk, self-screening, mobile app, older person

## Abstract

**Background:**

Recent falls prevention guidelines recommend early routine fall risk assessment among older persons.

**Objective:**

The purpose of this study was to develop a Falls Screening Mobile App (FallSA), determine its acceptance, concurrent validity, test-retest reliability, discriminative ability, and predictive validity as a self-screening tool to identify fall risk among Malaysian older persons.

**Methods:**

FallSA acceptance was tested among 15 participants (mean age 65.93 [SD 7.42] years); its validity and reliability among 91 participants (mean age 67.34 [SD 5.97] years); discriminative ability and predictive validity among 610 participants (mean age 71.78 [SD 4.70] years). Acceptance of FallSA was assessed using a questionnaire, and it was validated against a comprehensive fall risk assessment tool, the Physiological Profile Assessment (PPA). Participants used FallSA to test their fall risk repeatedly twice within an hour. Its discriminative ability and predictive validity were determined by comparing participant fall risk scores between fallers and nonfallers and prospectively through a 6-month follow-up, respectively.

**Results:**

The findings of our study showed that FallSA had a high acceptance level with 80% (12/15) of older persons agreeing on its suitability as a falls self-screening tool. Concurrent validity test demonstrated a significant moderate correlation (*r*=.518, *P*<.001) and agreement (k=.516, *P*<.001) with acceptable sensitivity (80.4%) and specificity (71.1%). FallSA also had good reliability (intraclass correlation .948; 95% CI .921-.966) and an internal consistency (*α*=.948, *P*<.001). FallSA score demonstrated a moderate to strong discriminative ability in classifying fallers and nonfallers. FallSA had a predictive validity of falls with positive likelihood ratio of 2.27, pooled sensitivity of 82% and specificity of 64%, and area under the curve of 0.802.

**Conclusions:**

These results suggest that FallSA is a valid and reliable fall risk self-screening tool. Further studies are required to empower and engage older persons or care givers in the use of FallSA to self-screen for falls and thereafter to seek early prevention intervention.

## Introduction

Falls among older persons are a major health and socioeconomic concern globally [1]. Fall prevalence ranged from 4.2% to 61% in Malaysian older persons in 2018 [2]. This range is similar to other older Asians in Japan and United Arab Emirates (18% to 57%) [3,4]. This could possibly stem from the similar research methodology in which history of falls in the past 12 months is commonly used in studies. In our earlier large-scale population-based longitudinal study Long-Term Research Grant Scheme—Toward Useful Ageing (LRGS TUA), we reported a retrospective and prospective fall prevalence of 15% to 18% and 27%, respectively, among Malaysian community-dwelling older persons [5-7]. Single and repeated fall incidence rates were 8.47 and 3.21 per 100 person-years, respectively [8]. In our local context, community-dwelling older persons are defined as older adults aged 60 years and above who are living independently in the community [9]. Identified risk factors associated with falls were arthritis, diabetes, urinary incontinence, decreased handgrip strength, higher BMI, and poor self-rated health [7], while the predictors consisted of a history of falls and decreased muscle strength for both occasional and recurrent falls [8].

Falls comprise multifactorial etiology, and they occur as a result of interactions between multiple intrinsic and extrinsic factors. Current evidence-based practice guidelines recommend early falls screening using a multifactorial fall risk assessment tool [1]. The combination of sociodemographic factors (gender, joint pain and cataract/glaucoma), self-rated multifactorial questionnaire (previous fall history and worrying about falls), and a physical performance test (Timed Up and Go [TUG]) in a fall risk model was proposed to identify fall risk among Malaysian community-dwelling older persons [6]. The combination of multifactorial evidence-based assessments is expected to be more robust compared to a single test [10]. There may be a potential to use an app-based fall risk multifactorial assessment.

Currently, there are several app-based fall risk assessment tools, including Lindera, Steady, and Aachen fall prevention apps. Lindera is a smartphone-based app designed to facilitate the health care professional (nursing staff) to perform a structured multifactorial fall risk assessment among older adults [11]. Lindera consists of a mobility test (TUG test) and fall risk–related questionnaire. It was found to have a pooled sensitivity of 93% and specificity of 58%, with an overall accuracy of 73% [11]. In Steady, 5 progressively more challenging mobility tests (30s balance and sit to stand tests ) and a medical history questionnaire were used to assess individual fall risk [12]. Steady was found to be valid and reliable in facilitating fall risk self-screening among older persons in home settings [13]. The Aachen fall prevention app has a combination of a balance test with simple questionnaire to improve fall risk awareness among older persons [14]. It has a sensitivity and specificity of 57% and 76.7%, respectively [14]. While Aachen and Steady were developed as fall self-screening tools, Lindera is used to assist health care professionals (nursing staff) in conducting multifactorial fall risk assessment among older persons.

Although multifactorial app-based fall risk assessment tools are currently available, they lack comprehensive information regarding their reliability and validity as self-assessment tools. Prior to the use of fall risk apps, their diagnostic accuracy in discriminating between fallers and nonfallers and predicting actual falls among at-risk older persons must be demonstrated. Existing fall risk assessment tools in clinical settings were found to have only moderate diagnostic accuracy [15]. Moreover, there is a need to develop a culturally specific fall risk assessment tool for the multiethnic Malaysian older population. Therefore, in this study, we aimed to develop the Fall Risk Screening App (FallSA) as a self-screening tool for assessing fall risk among Malaysian older persons using the combination of questionnaires and physical tests. Thereafter, we determined its acceptance, validity, reliability, discriminative ability, and predictive validity. We hope that with the early self-screening tool FallSA we are able to empower and engage older persons or their caregivers to be aware of falls and adopt fall prevention behavior.

## Methods

### Study Design

This study regarding FallSA was divided into 4 phases comprising (1) development, (2) acceptance among older persons, (3) concurrent validity and test-retest reliability, and (4) discriminative ability and predictive validity.

Ethical approval was obtained from the secretariat for research of ethics of Universiti Kebangsaan Malaysia (UKM 1.5.3.5/244/NN-060-2013 and UKM PPI/111/8/JEP-2018-559). Prior to all studies, participants were given information about the study and were required to provide informed verbal consent.

### Phase 1: Development

Prior to the development of FallSA, a literature review was conducted to identify current gaps in the literature regarding fall risk mobile screening apps. Next, several group discussions and meetings were conducted to identify the intended features, interface, and design to meet the functional needs of older persons. After which, preparation of the proposed product features and design using international guidelines for ease of use and usability of graphic user interface for older persons (ISO/IEC 2001) was done.

FallSA was developed based on a model established from our team’s large-scale study report (Neuroprotective Model for Healthy Longevity), which was designed to evaluate the magnitude of cognitive decline and its risk factors through comprehensive multifactorial assessment [16]. The significant predictive fall risk factors were used for the fall risk calculations in FallSA [5]. This fall risk model included the combination of sociodemographic information (gender, joint pain, and cataract/glaucoma), self-rated multifactorial questionnaire (previous fall history and worrying about falls), and physical performance test (TUG test) [5]. TUG normative values from the study by Ibrahim et al [5] were used to generate graphs in comparing user TUG test with 50th population norms-based age groups and gender. An instructional video for TUG test performance was provided within the FallSA app. The step-by-step procedure to perform the TUG self-test was based on the original version by Podsiadlo and Richardson [17]. Several modifications were done later to fulfill the self-screening feature of a mobile app. The designs of the icons were obtained from the readily available Google Advanced Image Search. While the icons were derived from an online source of free icons [18] and made readily available under the terms of the end user license agreement, their selection and modification were based on international guidelines (ISO/IEC Guide 71:2001 [E]). The icons were modified using Microsoft Paint, Microsoft Photos, and the Microsoft Snipping Tool. With respect to the guidelines, all text was written in black on a white-colored background to achieve maximum contrast. An adequate icon size was ensured for easy system navigation and indication of the current interface.

The input button, navigation, and arrangements were developed following the same guidelines. The navigation buttons of FallSA were structured in a rectangle with some simple instruction text at the bottom of the interface; it is displayed as an icon shape on the current user section. In addition, colored navigation buttons were employed in questions sections with a selection of yes in green and no in red. In order to increase ease of use among older persons, a simple navigation system flow was used by having only 2 main selections: new user and current user. The app features for wording, questions, and instructions were bilingual in English and Malay languages, which are commonly used among Malaysian population.

Prior to the use of FallSA, participants were shown an informed consent on data protection policy at the beginning of the app interface. Personal information or data were only saved in the user’s device and could only be assessed by the user and researcher. It is also noteworthy that the information stored in FallSA will be handled in a similar way to hard copies of medical records, as declared in the Data Protection Disclaimer and Laws of Malaysia Act 709 and Personal Data Protection Act 2010. Since FallSA is a self-screening mobile app for identifying fall risk and it does not provide any diagnosis or suggestion to change current medical dosages, it is not categorized as a medical device, which would require an approval from health authorities.

FallSA was developed by a freelance mobile software developer with more than 3 years’ experience in a related field, based on the proposed product features and design of the researcher team. The development of FallSA was conducted according to the waterfall software development process using agile principles for better quality, time, and cost-effective mobile app development.

### Phase 2: User Acceptance Testing

FallSA user acceptance testing was tested in the real world by 15 older persons.

#### Participants

A convenience sampling method was used to recruit the participants from 2 senior citizen clubs via letters of invitation. Participants were community-dwelling older persons aged 60 years and over and were from the main ethnicities in Malaysia (Malay, Chinese, and Indian). Inclusion criteria included being 60 years and above, able to comprehend the Malay or English language, able to use a smartphone, and able to ambulate with or without assistive devices with minimal supervision. Older persons with acute illnesses (unstable heart diseases and vestibular disorders) were excluded.

#### Procedure and Instrumentation

Participants were given information regarding the procedures and consent to participate in this study. Participant level of smartphone proficiency was determined verbally before conducting the study. Participants were presented with FallSA on a researcher’s mobile, and they were asked to open the app and follow its instructions with minimal guidance from the researcher. They then needed to complete both levels of navigation (new user and return user) in order to finish a real-life environment response of the software system. The FallSA test took approximately 15 minutes to complete. Upon completion, participants were required to provide feedback using a series of closed- and open-ended questions regarding their understanding of FallSA in relation to its features and design: (1) color contrast, (2) graphics or illustration, (3) font size, (4) presentation of instructional video, and (5) overall FallSA suitability. A modified version of a technology acceptance model survey was also used. The technology acceptance model is rated on 7-point Likert scale and has a high internal reliability (Cronbach 𝛼=.96) and positive correlation between each determinant (perceived usefulness, perceived ease of use, intention and attitude toward use of mobile technology) [19].

### Phase 3: Concurrent Validity And Test-Retest Reliability

Phase 3 was a cross-sectional study to determine the concurrent validity and test-retest reliability of FallSA among community-dwelling older persons.

#### Participants

Participants were recruited among community-dwelling older persons at two other senior citizen clubs. A total of 91 community-dwelling older persons participated in this study. The sampling method and its inclusion and exclusion criteria were similar to the phase 2 study.

#### Procedure and Instrumentation

In order to identify the concurrent validity of FallSA, the researcher validated FallSA with an existing fall risk assessment tool. To date, there is no gold standard assessment for fall risk [17]. Thus, the Physiological Profile Assessment (PPA) was selected because of its robustness in identifying fall risk among older persons by assessing their impairments in main physiological measurements irrespective of health conditions. Despite this, studies found that the PPA had 75% accuracy and moderate reliability of ≥0.50 in identifying risk of falls among older persons [20]. A short-form PPA comprising 5 questions was used to assess fall risk and was based on the composite (z) score calculated using online software. Fall risk in the PPA is categorized as follows: (1) very low, (2) low, (3) mild, (4) moderate, (5) marked, and (6) very marked [20].

Upon screening based on inclusion and exclusion criteria, participants were briefed on the assessments and informed consent was given. Next, participants proceeded with anthropometry measurements (height and weight), followed by collecting their sociodemographic data, fall history, and associated characteristics. Participants were then asked to use the latest version of the FallSA with minimal guidance from the trained enumerator. Participants selected their language preference (Malay/English) and then could proceed to the first section, registration of sociodemographic information. Upon completion of the registration section, participants were directed to the page on physical performance test instruction. Participants were asked to use all methods of instruction: wording, audio, and video. Following this, the interface changed to the physical performance test, with the start and stop timer being a big red button for ease of use among older persons. Two trials were attempted, and the mean was calculated. Next, participants were directed to 4 closed-ended questions regarding fall risk. Report of the overall result was presented upon completion with the whole assessment taking approximately 15 minutes to accomplish.

Participants were given a break of at least 15 minutes before proceeding to the short-form PPA. Trained physiotherapists with more than 1 year of experience were employed to administer the PPA. Participants were asked to perform the 5 tests included in the PPA: (1) edge contrast sensitivity (vision), (2) peripheral sensation (proprioception), (3) finger press (reaction time), (4) standing on the medium-density foam rubber mat (body sway), and (5) knee extension (lower limb strength). On average, the PPA was completed within 30 minutes.

After a 15-minute break upon completion of the PPA, participants repeated the FallSA test to determine its reliability. After verifying their identity card number and date of birth, participants directly proceeded to the physical performance test. Each participant took 10 minutes to complete this FallSA test.

### Phase 4: Discriminative Ability and Predictive Validity

Phase 4 was a cross-sectional study to identify the discriminative ability of FallSA followed by a 6-month prospective follow-up to examine its predictive validity in identifying risk of falls among the older persons in Peninsular Malaysia.

#### Participants

A total of 610 community-dwelling older persons from Peninsular Malaysia (Johor, Selangor, Perak, and Kelantan) participated in this cross-sectional validation study. Participants were selected using a multistage random sampling method. Participants aged 60 years and over able to ambulate independently with or without assistive devices were included in this study, whereas those unable to comprehend and follow instructions, having severe medical conditions, having severe vision or hearing impairments, or having cognitive impairments (dementia or depression) were excluded from this study.

#### Procedure and Instrumentation

Participant sociodemographic data, fall history, and medical condition were obtained. FallSA assessment was performed after a demo and trial session to assess their risk of fall. In the discriminative study, FallSA scores were compared among fallers and nonfallers according to their past 1-year fall history data. A faller was defined as someone who had one or more falls in the past year and nonfaller as someone without any falls [21].

Next, the validation study was conducted whereby participants were provided with a fall diary to document fall incidence monthly for a period of 6 months. In addition, participants were contacted via phone monthly to obtain their fall incidence and any other feedback and to remind participants about documenting their falls if any occur. The diaries were collected after 6 months to determine the predictive validity of FallSA. At the end of the 6-month follow-up, participants who had a risk of falls were advised verbally to seek further fall risk assessment and management at their primary health care settings.

### Statistical Analysis

The data were analyzed using SPSS (version 22.0, IBM Corp). Researchers performed the normality test for continuous variables in advance by using a Shapiro-Wilk test, Kolmogorov-Smirnov test, kurtosis, skewness ratio, histogram, stem and leaf, or box plot.

Descriptive analysis was performed on the sociodemographic profile. In phase 2, a Pearson correlation analysis was conducted to determine the acceptance level of FallSA among Malaysian community-dwelling older persons. In phase 3, the concurrent validity of FallSA was analyzed using Spearman correlation, kappa for agreement, sensitivity, and specificity. The test-retest reliability was conducted to identify internal consistency (Cronbach alpha), and the intraclass correlation (ICC) and Bland-Altman agreement were determined between 2 trials of the physical performance test. In the final phase, the discriminative ability of FallSA was analyzed using an independent *t* test, and the receiver operating characteristic (ROC) curve was used to identify the predictive validity of FallSA, with its sensitivity, specificity value, and cutoff point.

## Results

### Phase 1: FallSA Final Version

**Figure 1:**
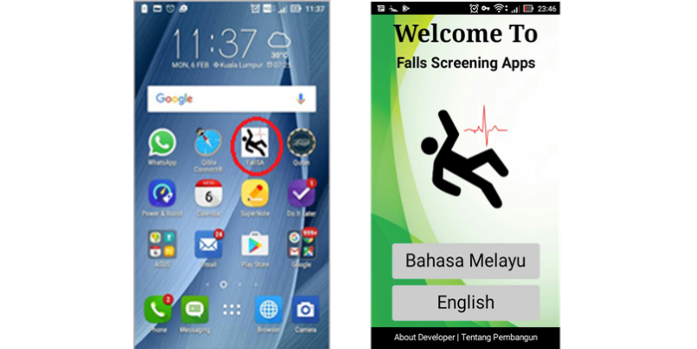
User initial navigation and language selection.

**Figure 2:**
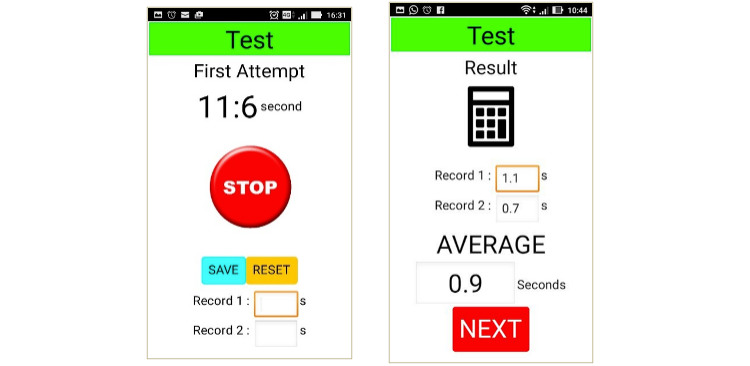
Physical Performance Test (TUG) results.

**Figure 3:**
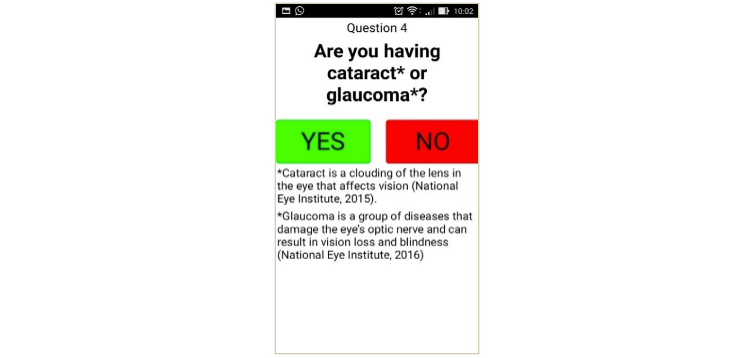
Sociodemographic and self-rated multifactorial questionnaire.

**Figure 4:**
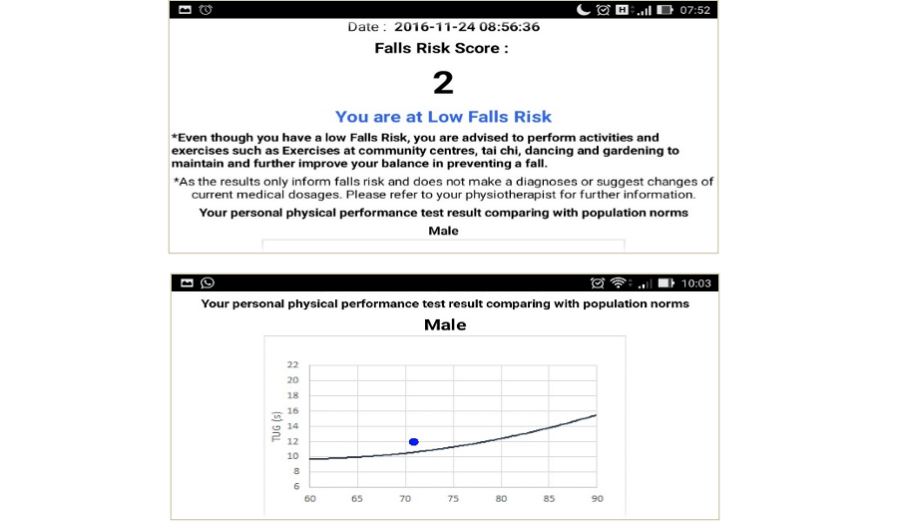
Fall risk reports.

The figures below depict the screen shots of FallSA (final version): selection of the Malay or English language, which are commonly used in the Malaysian population (Figure 1), TUG self-conduct test results (Figure 2), items from the sociodemographic and multifactorial questionnaire (Figure 3), and fall risk report (Figure 4).

### Phase 2: User Acceptance Testing Results

Table 1 depicts the sociodemographic data and user acceptance survey of FallSA based on gender. The results of our study found that more than 90% (14/15) of participants were able to comprehend the contents of FallSA and the instructional video provided in the app and agreed with its suitability of graphics and color combinations. However, approximately 30% (3/15) of participants reported that the font size used in FallSA was not suitable for older persons. Overall, 80% (12/15) of participants found that FallSA is suitable as a self-screening mobile app to identify fall risk among Malaysian community-dwelling older persons.

Participants listed 4 aspects in the FallSA app that were found to be not suitable. First was the physical performance test as the users were required to learn and understand how to perform the TUG test independently. However, most of the participants agreed that the TUG test was easy to use after minimal guidance. Second, participants found the font, especially in the disclaimer interface section, to be small for them. Participants stated that they had difficulty reading the instructions and information when using the app. Third, the system hanged occasionally, probably due to an unstable server and nonsynchronization between the apps and online database. Last, 15% (3/15) of participants requested more interesting graphics or illustrations. Technology Acceptance Model (TAM) survey results showed the presence of a high correlation (0.70 and above) between all determinants (perceived usefulness, perceived ease of use, intention and attitude towards usage of mobile technology).

**Table 1:** Sociodemographic information and user evaluation of different aspects of FallSA based on gender.

Characteristic				Males (n=6), n (%)		Females (n=9), n (%)	Total (n=15), n (%)
**Sociodemographic**							
	**Age (years)**						
		60-69	4 (36)		7 (64)		11 (73)
		≥70	2 (50)		2 (50)		4 (27)
	**Race**						
		Malay	2 (29)		5 (71)		7 (47)
		Chinese	3 (60)		2 (40)		5 (33)
		Indian	1 (33)		2 (67)		3 (20)
	**Education level**						
		None	3 (75)		1 (25)		4 (27)
		Primary	2 (33)		4 (67)		6 (40)
		Secondary	—^a^		3 (100)		3 (20)
		Tertiary	1 (50)		1 (50)		2 (13)
	**Marital status**						
		Married	2 (25)		6 (75)		8 (53)
		Widowed	2 (50)		2 (50)		4 (27)
		Divorced	1 (50)		1 (50)		2 (13)
		Single	1 (100)		—		1 (7)
**User evaluation of the different aspects of FallSA**							
	**Contents**						
		Understand	5 (36)		9 (64)		14 (93)
		Did not understand	1 (100)		—		1 (7)
	**Suitability of graphics**						
		Suitable	5 (36)		9 (64)		14 (93)
		Not suitable	1 (100)		—		1 (7)
	**Color combination**						
		Suitable	6 (43)		8 (57)		14 (93)
		Not suitable	—		1 (100)		1 (7)
	**Font size**						
		Easy to read	4 (40)		6 (60)		10 (67)
		Hard to read	2 (40)		3 (60)		5 (33)
	**Instructional video**						
		Easy to understand	6 (40)		9 (60)		15 (100)
		Hard to understand	—		—		—
	**Overall suitability of FallSA**						
		Suitable	5 (42)		7 (58)		12 (80)
		Not suitable	1 (33)		2 (67)		3 (20)

^a^Not applicable.

### Phase 3: Concurrent Validity (Against PPA) and Test-Retest Reliability Results

Participant characteristics are shown in Table 2.

The concurrent validity results between FallSA and PPA are shown in Table 3. PPA results were categorized in dichotomous data using cutoff points (high and low risk of fall). There was a significant moderate correlation (*r*=.518, *P*<.001) found between FallSA and PPA. All test parameters area under the ROC curve and Cohen kappa were statistically acceptable with sensitivity and specificity at 80.4% and 71.1%, respectively. There was a stronger correlation between FallSA and PPA in males (*r*=.538, *P*<.001) compared to females (*r*=.502, *P*<.001), with a higher sensitivity value of 88.9%. In addition, older persons with higher (secondary and tertiary) education (*r*=.427, *P*<.001) had a lower correlation between FallSA and PPA compared to those with lower (none and primary) education level (*r*=.511, *P*<.001).

**Figure 5:**
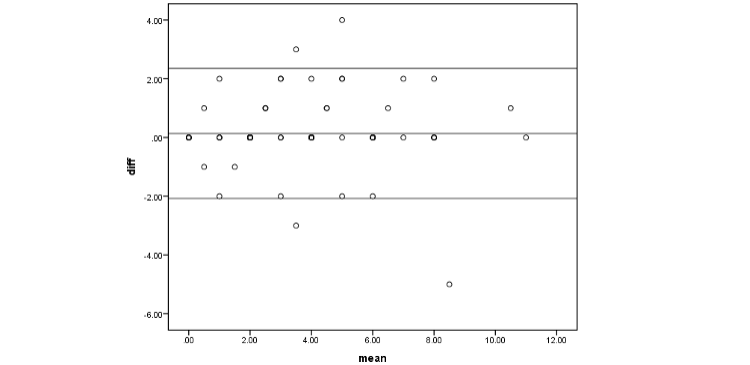
Limit of agreement.

As for test-retest reliability, there was a significant high reliability between repeated FallSA tests (*P*<.001; ICC .948, 95% CI .921-.966) as shown in Table 4. There was high agreement with small mean differences and narrow limits of agreement between repeated FallSA scores (Figure 5).

**Table 2:** Sociodemographic data of the participants based on gender.

Characteristics		Males (n=40), n (%)	Females (n=51), n (%)	Total (n=91), n (%)
**Age (years)**				
	60-69	19 (32)	41 (68)	60 (66)
	≥70	21 (68)	10 (32)	31 (34)
**Race**				
	Malay	29 (47)	33 (53)	62 (68)
	Chinese	9 (36)	16 (64)	25 (28)
	Indian	2 (50)	2 (50)	4 (4)
**Education level**				
	None	—^a^	2 (100)	2 (2)
	Primary	8 (53)	7 (47)	15 (17)
	Secondary	19 (41)	27 (59)	46 (51)
	Tertiary	13 (46)	15 (54)	28 (31)
**Marital status**				
	Married	37 (56)	29 (44)	66 (73)
	Widowed	1 (6)	17 (94)	18 (20)
	Divorced	—	2 (100)	2 (2)
	Single	2 (40)	3 (60)	5 (6)

^a^Not applicable.

**Table 3:** Concurrent validity of FallSA against Physiological Profile Assessment (PPA).

Variables			*r* value		Kappa		Sensitivity (%)		Specificity (%)		ROC^a^
**Gender**											
	Males	.538		.494		88.9		74.2		0.749	
	Females	.502		.429		70.7		90.0		0.790	
**Education level**											
	Low	.511		.628		81.8		83.3		0.902	
	High	.427		.489		80.0		69.2		0.776	
Total score			.518		.516		80.4		71.1		0.794

^a^ROC: receiver operating characteristic.

**Table 4:** Reliability of the FallSA total score.

Characteristic			Cronbach alpha		ICC^a^		95% CI		SEM^b^
Total score FallSA			.948		.948		.921-.966		1.11
**Gender**									
	Male	.920		.920		.849-.958		0.98	
	Female	.929		.929		.875-.959		1.21	
**Education**									
	Low	.974		.974		.929-.991		0.88	
	High	.942		.942		.908-.963		1.15	

^a^ICC: intraclass correlation.

^b^SEM: standard error of the mean.

### Phase 4: Discriminative Ability and Predictive Validity Results

A total of 1005 community-dwelling older persons aged 60 years and above within Peninsular Malaysia (Selangor, Perak, Johor, and Kelantan states) were screened for the inclusion criteria; 395 were excluded due to having a score ≥5 on the Geriatric Depression Scale, a score ≤21 on the Mini Mental State Examination, or failing to complete the FallSA test. The sociodemographic data of the participants is shown in Table 5. Participants with a past history of falls had significantly higher FallSA scores (7.33 [SD 1.77]) at baseline as compared to those without any falls (4.55 [SD 1.86]; *P*<.001). This indicates the discriminative ability of FallSA.

After 6 months, 74.4% (454/610) of participants were successfully followed up via fall diary and monthly phone calls. The 26.6% (156/610) who were dropouts were excluded from the follow-up analysis. About 14.5% (66/454) of the community-dwelling older persons had a fall after the 6-month follow-up. FallSA scores were compared with actual falls reported after the 6-month follow-up; 3% (3/66) of participants categorized as low risk and 18% (63/66) of participants categorized as at-risk experienced a fall.

The cutoff, sensitivity and specificity values, and positive and negative likelihood ratios of FallSA are presented in Table 6. The fall risk score of FallSA ranges from 0 to 11. The results of this study suggest a FallSA cutoff score of >5 is the best predicted cutoff point of fall among older persons. With this cutoff, the sensitivity and specificity values of the FallSA score were 81.82% (95% CI 70.4-90.2) and 63.92% (95% CI 58.9-68.7), respectively, with a positive likelihood ratio of 2.27, meaning those community-dwelling older persons were 2.27 times more likely to fall compared to those who scored ≤5.0. The Youden index shown in analysis was 0.47.

**Figure 6:**
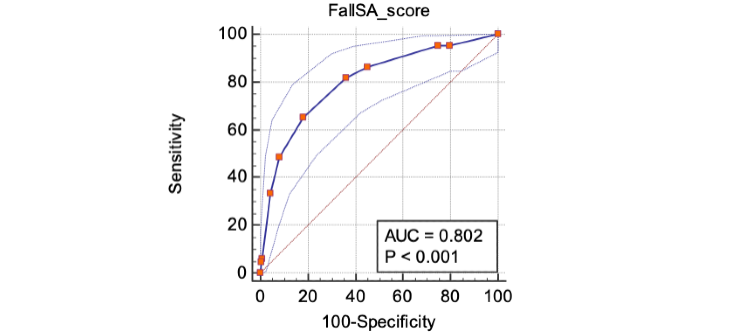
Receiver operating characteristic of FallSA based on the 6-month follow-up.

The ROC of FallSA is shown in Figure 6. With an average area under the curve (AUC) of 0.802, FallSA is demonstrated to have a good discriminative ability. An excellent result for AUC is indicated if close to 1 (0.8-0.9) [22].

**Table 5:** Baseline sociodemographic data classified based on fallers and nonfallers.

Variables		Total (n=610)		Fallers (n=111; 18.2%)		Nonfallers (n=499; 81.8%)		*P* value
Age, mean (SD)		71.78 (4.7)		72.01 (4.6)		71.73 (4.8)		.58
MMSE^a^, mean (SD)		26.48 (2.4)		26.43 (2.4)		26.49 (2.4		.82
GDS^b^, mean (SD)		1.85 (1.3)		1.96 (1.3)		1.83 (1.3)		.32
PASE^c^, mean (SD)		125.76 (54.4)		125.49 (50.8)		125.82 (55.3)		.95
**Gender, n (%)**		—^d^		—		—		.001
	Male	332 (54.4)	45 (13.6)		287 (86.4)		—	
	Female	278 (45.6)	66 (23.7)		212 (76.3)		—	
**Race, n (%)**		—		—		—		.85
	Malay	361 (59.2)	65 (18.0)		296 (82.0)		—	
	Chinese	218 (35.7)	40 (18.3)		178 (81.7)		—	
	Indian	31 (5.1)	6 (19.4)		25 (80.6)		—	
**Education level, n (%)**		—		—		—		.79
	None	63 (10.3)	10 (15.9)		53 (84.1)		—	
	Primary	306 (50.2)	60 (19.6)		246 (80.4)		—	
	Secondary	199 (32.6)	34 (17.1)		165 (82.9)		—	
	Tertiary	31 (5.1)	7 (22.6)		24 (77.4)		—	
	Other	11 (1.8)	0		11 (100)		—	
**Chronic illness, n (%)**		—		—		—		—
	Hypertension	83 (13.6)	18 (21.7)		65 (78.3)		.83	
	Diabetes	163 (26.7)	34 (20.9)		129 (79.1)		.84	
	Heart disease	62 (10.2)	15 (24.2)		47 (75.8)		.55	
	Arthritis	209 (34.3)	44 (21.1)		165 (78.9)		.15	
**Falls history in past 12 months, n (%)**		—		—		—		.001
	No falls	499 (81.8)	0		499 (100)		—	
	1 fall	68 (11.1)	68 (100)		0		—	
	≥2 falls	43 (7.1)	43 (100)		0		—	
**Medication, n (%)**		—		—		—		.47
	< meds	440 (72.1)	75 (17.0)		365 (83.0)		—	
	≥ meds	170 (27.9)	36 (21.2)		134 (78.8)		—	
**Eye problems, n (%)**		—		—		—		.56
	Yes	105 (17.2)	17 (16.2)		88 (83.8)		—	
	No	505 (82.8)	94 (18.6)		411 (81.4)		—	
**Use of assistive devices, n (%)**		—		—		—		.05
	Yes	31 (5.1)	11 (35.5)		20 (64.5)		—	
	No	579 (94.9)	100 (17.3)		479 (82.7)		—	
FallSA score, mean (SD)		5.05 (2.15)		7.33 (1.77)		4.55 (1.86)		.001
**FallSA Fall Risk, n (%)**		—		—		—		.001
	Low risk	132 (21.6)	1 (0.8)		131 (99.2)		—	
	At risk	478 (78.4)	110 (23.0)		368 (77.0)		—	

^a^MMSA: Mini Mental State Examination.

^b^GDS: Geriatric Depression Scale.

^c^PASE: Physical Activity Scale for Elderly.

^d^Not applicable.

**Table 6:** Criterion values and coordinates of the receiver operating characteristic curve in the FallSA fall risk score.

Criterion	Sn^a^	Sp^b^	+LR^c^	–LR^d^	PPV^e^	NPV^f^
≥2	100	0	1	—^g^	14.5	—
>2	95.45	20.36	1.20	0.22	16.9	96.3
>3	95.45	25.26	1.28	0.18	17.8	97.0
>4	82.35	54.90	1.91	0.25	24.6	95.9
>5^h^	81.82	63.92	2.27	0.28	27.8	95.4
>6	65.15	81.70	3.56	0.43	37.7	93.2
>7	48.48	91.75	5.88	0.56	50.0	91.3
>8	33.33	95.62	7.61	0.70	56.4	89.4
>9	6.06	99.23	7.84	0.95	57.1	86.1
>10	4.55	99.48	8.82	0.96	60.0	86.0
>11	0	100	—	1	—	85.5

^a^Sn: sensitivity.

^b^Sp: specificity.

^c^LR+: positive likelihood ratio.

^d^LR–: negative likelihood ratio.

^e^PPV: positive predictive value.

^f^NPV: negative predictive value.

^g^Not applicable.

^h^Cutoff score.

## Discussion

### Principal Findings

In our study, we successfully developed an accepted and validated mobile app, FallSA, that has the potential to identify a risk of falls among a multiethnic Malaysian older population. The FallSA score manifested a moderate to high discriminative ability and predictive validity in classifying fallers and nonfallers and predicting falls among older persons. A cutoff score of >5.0 is recommended in our FallSA predictive results. With this cutoff, FallSA had a positive likelihood ratio of 2.27, pooled sensitivity of 82%, and specificity of 64% with an average AUC of 0.802. However, only 4.5% of older persons screened to have a low fall risk using FallSA had experienced falls in the previous 6 months in our study. This suggests that the present sensitivity and specificity of the FallSA test would be more useful for nonfallers.

Our findings showed that 80% of the participants agreed that FallSA is suitable for use as a self-screening tool among Malaysian community-dwelling older persons. Similarly, the Aachen fall prevention app [14] was found to be well accepted and useful when offered in the mobile stores. These results suggest it is possible to facilitate the use of a fall risk mobile app among older persons and their caregivers to self-screen for fall risk. FallSA as a mobile health technology with automated reports would be useful as a self-administered fall risk assessment tool. Such tools can be administered at home or in community settings and save time [23].

There were several rounds of adjustments in the development stages (requirement design, implementation, and verification) of the FallSA app that included changes in concept, design, graphics, contents, navigation flow, and technical and language corrections. In the user acceptance stage, the design of FallSA was well accepted and older persons had a positive attitude toward FallSA’s adoption with some minor use issues. We addressed all the highlighted issues in the acceptance test before proceeding to conduct reliability and validity tests. For example, font size in FallSA was increased within the limitations of smartphone display size. In addition, we provided bilingual and simplified video instructions for the TUG test. Increasing the number of icons used in the graphic user interface and rectifying problems in the coding system that caused the system to hang during use were also addressed.

Upon addressing participant feedback regarding FallSA, we examined the concurrent validity of FallSA against PPA among 91 community-dwelling older persons. To date, there is no gold standard assessment tool to measure fall risk. However, PPA was identified as the most comprehensive evidence-based practice fall risk assessment tool available. Our study results showed that there is a significant moderate correlation between FallSA and PPA fall risk measurements (*P*<.001, k=0.875, *r*=.518), with good sensitivity (80.4%) and specificity (71.1%) and good ROC (0.794) association. The plausible explanation of this moderate correlation could be because PPA assessed different aspects of fall risk factors (proprioception, contrast sensitivity, postural sway, reaction time, and lower limb strength) objectively. Besides the TUG test in FallSA, the rest are based on questionnaires and are subjective. However, these findings support the concurrent validity of FallSA against PPA, suggesting that FallSA is able to identify fall risk in Malaysian community-dwelling older persons. Although only a moderate correlation was found between FallSA and PPA, it is sufficient and in line with the use of FallSA as an early fall risk self-screening tool.

Compared to the concurrent validity of other fall screening tools such as the Fall Risk Questionnaire [24] and Austin Health Falls Risk Screening Tool [25], FallSA’s concurrent validity is slightly lower. This can be speculated as we only included 4 significant predictor variables to keep FallSA simple for self-use in older persons. It is also noteworthy that we tested its concurrent validity using comprehensive fall risk assessment tool rather than against another questionnaire. Furthermore, FallSA was designed to support an early screening that can be preceded upon inquiring for further comprehensive clinical assessments by trained health care professionals.

Test-retest analysis of first and second FallSA score results showed that all parameters had an excellent test-retest reliability with Cronbach 𝛼=.948 (ICC .948, 95% CI .921-.966; SEM 1.11), suggesting FallSA is consistent in assessing fall risk among older persons. In addition, a good agreement was demonstrated with Bland-Altman analysis, having a small mean difference and narrow limits of agreement between the first and second FallSA assessments.

The fall prevalence demonstrated in our phase 4 study was 18.2%, which corresponded to earlier local studies conducted among community-dwelling older persons in Malaysia (15% to 18%) [5,6]. However, this value is much lower compared to prevalence rates on a global scale, which are found to be 12% to 63% [26]. This variation could be possibly due to having active and younger older persons in the local studies. Further, this study was conducted among older persons in the community, of which the prevalence is expected to be lower than clinical settings or in residential homes [27].

There was a moderate to high discriminative ability and predictive validity in discriminating fallers and nonfallers and predicting falls among older persons using FallSA. The pooled predictive sensitivity (82%) and specificity (64%) of FallSA are comparable to the Aachen fall prevention app (sensitivity 57.0%; specificity 76.7%) in discriminating between fallers and nonfallers. It is noteworthy that the FallSA predictive validity was conducted prospectively based on actual falls, while the Aachen fall prevention app used its primary outcome in a cross-sectional manner. Using a FallSA cutoff score of >5.0, 95.5% of falls after the 6-month follow-up among community-dwelling older persons were predicted. The number of false negative results can be reduced along with a lower negative likelihood ratio and further reinforced with a higher sensitivity value [28]. Only 4.5% of older persons screened to have low fall risk using FallSA had experienced falls in the previous 6 months in our study.

Comparably, the AUC (0.84) and sensitivity value (93%) of Lindera were much higher than FallSA. This discrepancy is possibly due to the variation in study methodology whereby the discriminative ability of Lindera was based on retrospective fall information, whereas for FallSA it was conducted prospectively. Moreover, FallSA is developed as a self-screening fall risk assessment tool to support the older persons as an early fall screening tool, while Lindera is specifically designed to assist health care professionals in clinical settings to identify fall risk among the older people. FallSA having a higher average sensitivity compared to specificity value is more suitable for early fall screening and prevention. This will allow a greater proportion of older persons to be screened and participate in fall prevention programs. This is in line to support the call for routine fall risk assessment and early management in the updated National Institute for Health and Care Excellence (NICE) fall prevention statements [1]. In terms of advanced diagnostic testing in clinical settings, a high specificity value is needed to avoid the unnecessary, costly, and tiring management among the nonfallers.

### Limitations and Strengths

FallSA has a higher sensitivity value when compared to traditional fall risk assessment using the TUG test. TUG test sensitivity as a fall risk assessment was reported to range from 30.5% to 67.5% [29,30]. As a stand-alone fall risk assessment tool, the sensitivity of TUG is lower as fall risk is multifactorial in nature. Moreover, although mobility and balance status could be assessed using TUG [31,32], it may not be comprehensive enough to determine the multiple interacting fall risk factors. This deficiency has been addressed in FallSA as the TUG test is combined with fall-related multifactorial questions in the fall risk calculation model.

The model used in FallSA was derived from both urban and rural community-dwelling older populations. Hence FallSA’s use among older persons living in institutions and with frailty will require adaptations to the present model used. Another limitation of FallSA is that it does not provide information for specific impairment areas for tailored interventions as PPA does. However, FallSA is meant for preliminary fall risk self-screening. Last, the duration of follow-up for FallSA may be relatively short in this study, and the changes of health status among older persons may not have been accounted for.

The main strength of FallSA is that it was developed systematically and followed by testing its acceptance concurrent validity, reliability, and discriminative and predictive validity. In addition, we used prospective fall monitoring to identify the predictive validity of FallSA. Note that our study comprised older persons from Peninsular Malaysia and all 3 main ethnicities (Malays, Chinese and Indians) and the results can be generalized to the entire community-dwelling older population.

Clinically, FallSA has the potential to be used as a self-screening, caregiver administered, or at primary health care settings as an early fall risk screening tool. This will assist in the annual fall risk screening among older adults as outlined in the NICE updated fall prevention guidelines [1]. Packaged as a mobile app, FallSA is accessible anywhere anytime and is simple, fast, and easy to administer. Hence, it is also suitable to be used for large-scale community-based fall screenings. Early fall detection can assist in targeting for early fall prevention interventions in older persons at risk of falls. However, FallSA could be improved by enhancing the information in the instructional video for the TUG test, upgrading it with an educational video of fall risk, and adding a sit to stand test with its normative values.

### Conclusions

In this study, we successfully developed a mobile app (FallSA) to identify fall risk among community-dwelling older persons that was accepted, valid, and reliable. FallSA is a short multifactorial assessment tool as it integrates sociodemographic, clinical, and physical fall risk factors. Although NICE updated fall prevention guidelines have recommended an annual fall risk screening, community-dwelling older persons in our local setting tend to visit primary health care settings more often. Since this test is self-administered, we suggest at least a biannual fall risk screening among older persons. To the best of our knowledge, FallSA is one of the most comprehensively tested fall risk self-assessment tools. FallSA has the potential to be used as one of the fall prevention strategies with the ultimate aim of maintaining independence and improving quality of life as long as possible among older persons. Future studies are required to empower and engage older persons or care givers in the use of FallSA to self-screen for falls and thereafter seek early prevention intervention.

## Abbreviations

AUC: area under the curve
FallSA: Falls Risk Screening App
ICC: intraclass correlation
LRGS: Long-Term Research Grant Scheme—Toward Useful Ageing
NICE: National Institute for Health and Care Excellence
PPA: Physiological Profile Assessment
ROC: receiver operating characteristic
TUG: Timed Up and Go
