# Correction: A Mobile App (FallSA) to Identify Fall Risk Among Malaysian Community-Dwelling Older Persons: Development and Validation Study

**DOI:** 10.2196/34368

**Published:** 2021-10-28

**Authors:** Devinder Kaur Ajit Singh, Jing Wen Goh, Muhammad Iqbal Shaharudin, Suzana Shahar

**Affiliations:** 1 Center for Healthy Ageing and Wellness Faculty of Health Sciences Universiti Kebangsaan Malaysia Kuala Lumpur Malaysia; 2 Faculty of Health Sciences Cawangan Pulau Pinang, Kampus Bertam Universiti Teknologi Majlis Amanah Rakyat Penang Malaysia

In “A Mobile App (FallSA) to Identify Fall Risk Among Malaysian Community-Dwelling Older Persons: Development and Validation Study” (JMIR Mhealth Uhealth 2021;9(10):e23663), the authors noted one error.

In the originally published manuscript, the name of author *Devinder Kaur Ajit Singh* was formatted incorrectly as the given name “Devinder” and surname “Kaur Ajit Singh.” This has now been corrected to the given names “Devinder Kaur Ajit” and surname “Singh.” The citation information for this author has also been corrected from “Kaur Ajit Singh D” to “Singh DKA.”

The correction will appear in the online version of the paper on the JMIR Publications website on October 28, 2021, together with the publication of this correction notice. Because this was made after submission to PubMed, PubMed Central, and other full-text repositories, the corrected article has also been resubmitted to those repositories.

